# Research Ethics Committees in Africa: Authors' Reply

**DOI:** 10.1371/journal.pmed.0040136

**Published:** 2007-03-27

**Authors:** Nancy E Kass, Adnan Ali Hyder, Ademola Ajuwon, John Appiah-Poku, Nicola Barsdorf, Dya Eldin Elsayed, Mantoa Mokhachane, Bavon Mupenda, Paul Ndebele, Godwin Ndossi, Bornwell Sikateyo, Godfrey Tangwa, Paulina Tindana

We thank Dr. Benatar [1] for pointing out that South Africa has two Fogarty-funded bioethics training programs: one that focuses primarily on providing short-term training to mid-career professionals from Southern Africa; and another that provides modular training in research ethics to professionals from the African continent. In addition, there are now several other Fogarty-funded training programs that either target African professionals exclusively or include African professionals, among others, in their programs (see http://www.fic.nih.gov/programs/training_grants/bioethics/index.htm). All of these programs share the goal of increasing professional capacity in bioethics and research ethics on the African continent.

Our own paper demonstrated that training even a small number of individuals can make a difference in changing policy and practice regarding research ethics in several institutions; that so many training efforts are now ongoing is a major step forward. Again, having more people teaching and discussing research ethics and starting and staffing research ethics committees will never itself guarantee that research with humans is more ethical, but it seems to be a critical first step. Capacity development for Africa still remains a challenge and worthy of increasing investments in global health.
